# KLF13 promotes porcine adipocyte differentiation through PPARγ activation

**DOI:** 10.1186/s13578-015-0016-z

**Published:** 2015-06-10

**Authors:** Shuzhong Jiang, Hongkui Wei, Tongxing Song, Yang Yang, Feng Zhang, Yuanfei Zhou, Jian Peng, Siwen Jiang

**Affiliations:** Department of Animal Nutrition and Feed Science, College of Animal Science and Technology, Huazhong Agricultural University, Wuhan, P. R. China; The Cooperative Innovation Center for Sustainable Pig Production, Wuhan, 430070 China; Key Laboratory of Swine Breeding and Genetics of the Agricultural Ministry, College of Animal Science and Technology, Huazhong Agricultural University, Hongshan, 430070 P. R. China

**Keywords:** KLF13, Adipocyte differentiation, PPARγ, Transcriptional regulation, Porcine, Mouse

## Abstract

**Background:**

Adipogenesis is tightly controlled by a complex network of transcription factors acting at different stages of differentiation. Kruppel-like factors (KLFs) as a family of zinc-finger transcription factors play diverse roles during cell differentiation and development in mammals.

**Results:**

In the present study, we showed that KLF13 acts as a key regulator regulating porcine adipocyte differentiation. The expression of KLF13 was markedly up-regulated during the early stage of porcine adipocyte differentiation, which was followed by expression of PPARγ. Porcine adipocyte differentiation was significantly attenuated by the addition of siRNA against KLF13, whereas overexpression of KLF13 resulted in enhanced porcine adipocyte differentiation. Using promoter deletion and mutation analysis, we identified a KLF13-binding site within −593/-577 region of the porcine *PPARγ* proximal promoter, indicating that KLF13 directly interacts with porcine *PPARγ* promoter. However, inhibition of KLF13 by siRNA did not impair mouse adipocyte differentiation. In addition, knockdown and/or overexpression of KLF13 in 3 T3-L1 cells all did not influence expression of *PPARγ2.*

**Conclusions:**

Collectively, our results suggest that KLF13 exist as a key pro-adipogenic transcription factor through transactivating *PPARγ* expression in porcine adipocyte differentiation, whereas no such effect was detected in mouse adipocyte differentiation.

**Electronic supplementary material:**

The online version of this article (doi:10.1186/s13578-015-0016-z) contains supplementary material, which is available to authorized users.

## Background

The adipose tissue is not only an organ which is responsible for energy storage in the form of lipids, and, also acts as a major endocrine organ [1]. In pigs, intramuscular fat (IMF) content is critical for various meat quality attributes such as muscle color, firmness, water-holding capacity and also important nutritional aspects is critical for meat quality [1]. This excess of adipose tissue can be the consequence of both increased fat-cell number (hyperplasia) and increased fat-cell size (hypertrophy) [2]. The number of adipocytes present in an organism is determined to a large degree by the adipocyte differentiation process. Thus, to further understanding of adipocyte differentiation of pig may provide valuable information in animal body development and increasing meat quality.

Adipocyte differentiation is controlled by a tightly regulated transcriptional cascade where the transcription factors activate or repress the expression of each other in a sequential manner [3]. At the center of transcriptional cascade network are the two principal adipogenic factors, PPARγ and C/EBPα, which induce expression of the gene program that leads to the mature adipocyte phenotype [4]. PPARγ in particular is considered the master regulator of adipocyte differentiation, and its activation is both necessary and sufficient for adipocyte differentiation [4]. PPARγ can promote adipogenesis in C/EBPα-deficient cells, but the converse has not been tested [5]. In addition, many early adipogenic factors of adipocyte differentiation, both negative and positive, exert their effects through modulation of expression or activity of PPARγ [6].

Kruppel-like factors (KLFs) are members of an emerging family of DNA-binding transcriptional regulators with crucial roles in development, differentiation and a number of other physiological cellular processes [7]. The distinguishing feature of the KLF family is the presence of three highly conserved classical Cys2/His2 zinc fingers [8]. These zinc fingers are located at the carboxyl terminus of the KLF proteins and enable KLFs to specifically bind to GC- and CACCC-boxes in regulatory regions of target genes [9]. In recent years, more and more members of KLF have been identified to be involved in adipogenesis [10]. And a series of experiments demonstrated that KLF proteins had different expression patterns during adipocyte differentiation and a total of 9 KLFs (KLF2, KLF3, KLF4, KLF5, KLF6, KLF7, KLF8, KLF9 and KLF15) had been proved to either promote or inhibit adipocyte differentiation [10].

Kruppel-like factor 13 (KLF13), previously designated as basic transcription element-binding protein-3 (BTEB3), was further identified to be important for development and other physiological functions. KLF13 has recently been identified as key regulator of heart development [11], cholesterol homeostasis [12], and inflammatory response [13]. However, to date, there has been no evidence regarding the functional role of KLF13 in adipocyte differentiaiton. In our previous study, we used RNA-Seq technique to identify that the expression of *KLF13* was induced during adipogenic differentiation of porcine stromal vascular cells [14]. In the present study, we proved that KLF13 functioned in porcine adipocyte differentiation through transactivating PPARγ, whereas no such effect was detected in mouse adipocyte differentiation.

## Results

### KLF13 promotes porcine adipocyte differentiation *in vitro*

**Fig. 1:**
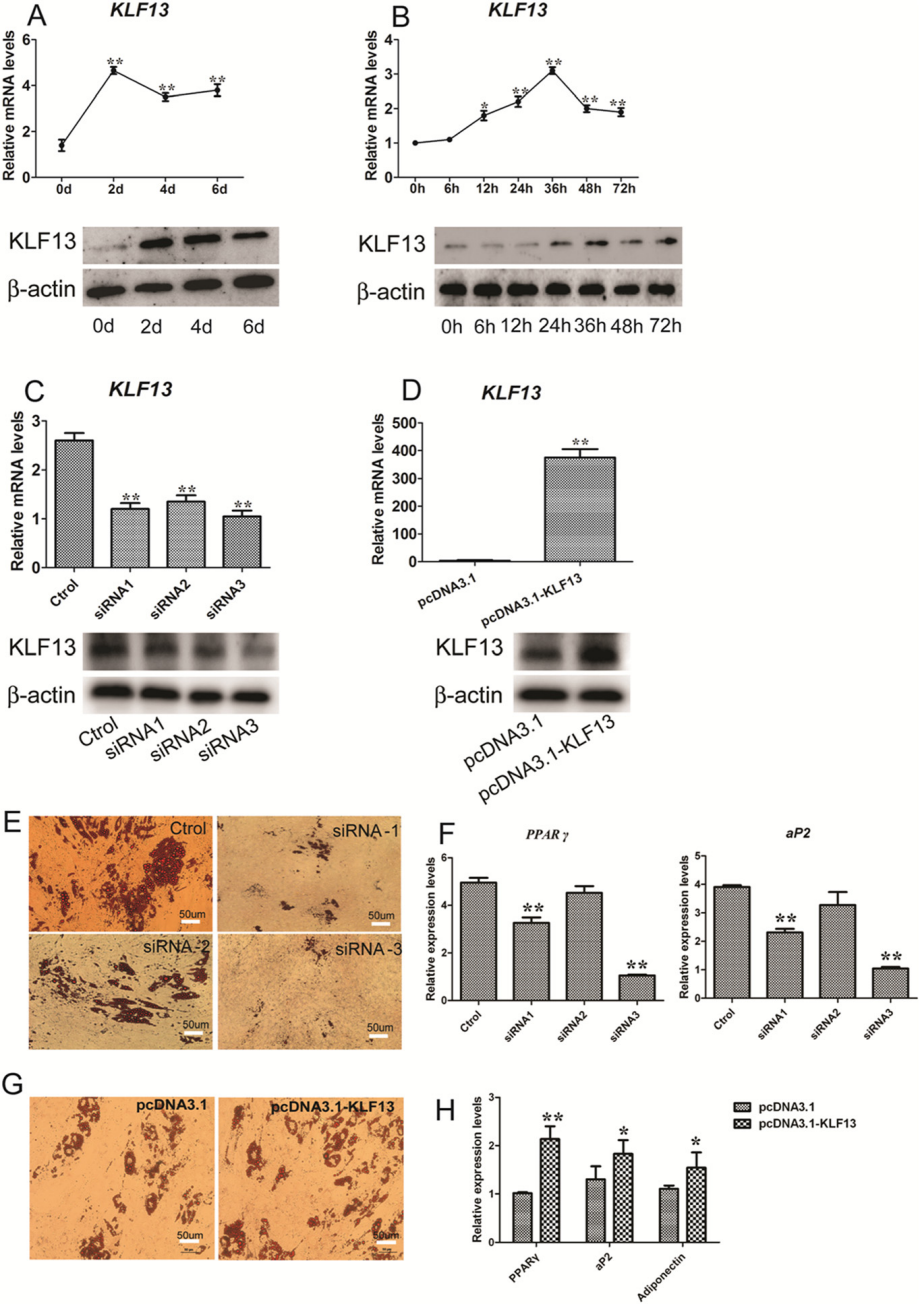
Expression and function of KLF13 in porcine ASVC. **a** The mRNA expression of *KLF13* in porcine ASVC during adipocyte differentiation. The mRNA level was determined by real-time PCR and normalized to β-actin mRNA. The numbers indicate the time points of differentiation induction. Results are expressed as means ± SD. (n = 3) **b** The mRNA and protein expression of KLF13 in porcine ASVC during the early stages of adipocyte differentiation. The mRNA level was determined by real-time PCR and normalized to β-actin mRNA. The protein level was determined by western blotting. The numbers indicate the time points of differentiation induction. Results are expressed as means ± SD. (n = 3). **c** The mRNA and protein expression of KLF13 in ASVC with the treatment of siRNA. siRNA1, siRNA2, and siRNA3 were three siRNA sequences (see Materials and methods for details). Ctrol indicates control siRNA sequence. Endogenous KLF13 mRNA was determined by real-time PCR in pre-induction. The protein level was determined by western blotting. Results are expressed as means ± SD. (n = 3). **d** The KLF13 mRNA level in ASVC transiently transfected with pcDNA3.1-KLF13 or empty vector. Endogenous KLF13 mRNA was determined by real-time PCR in pre-induction. The protein level was determined by western blotting. Results are expressed as means ± SD. (n = 3). **e** Blocked ASVC adipocyte differentiation by KLF13 knockdown. ASVC were treated with KLF13 siRNA at about 70 % confluence. After 24 h, the cells were induced to adipogenic differentiation. On day 8, the cell monolayer was stained with Oil-red O. **f** The mRNA expression of *PPARγ* and *aP2* in KLF13-knockdown ASVC were detected by real-time PCR on day 8 after adipogenic induction. Results are expressed as means ± SD. (n = 3). **g** Overexpression of KLF13 resulted in enhanced differentiation. ASVC were treated with KLF13 overexpression plasmid at about 70 % confluence. After 24 h, the cells were induced to adipogenic differentiation. On day 8, the cell monolayer was stained with Oil-red O. **h** The mRNA expression of *PPARγ*, *aP2* and *Adiponectin* in KLF13-overexpression ASVC were detected by real-time PCR on day 8 after adipogenic induction. Results are expressed as means ± SD. (n = 3) *P < 0.05, **P < 0.01

To detect whether KLF13 was related to porcine adipocyte differentiation, we isolated porcine primary ASVC and induced these cells for adipogenic differentiation. We analyzed the expression of *KLF13* during ASVC adipogenic differentiation induced by the standard IBMX, dexamethasone, and insulin (MDI) cocktail. The Real-time PCR analysis showed that *KLF13* mRNA significantly increased after MDI induction, reaching a maximum at day 2, and protein expression of KLF13 with similar patterns (Fig. 1a). This result indicated that KLF13 expression is induced at the early stage of ASVC adipogenic differentiation. Furthermore, we detected the expression of KLF13 during the early course of ASVC adipogenic differentiation. The result showed that the expression of KLF13 mRNA and protein significantly increased from 12 h of the MDI induction and peaked at 36 h post-induction (Fig. 1b). Furthermore, we detected the expression of *KLF13* during the early stage of porcine MSVC and DFAT cells adipogenic differentiation. The results showed that the expression patterns of *KLF13* during the early stage of porcine MSVC and DFAT cells adipogenic differentiation were similar to that of expression pattern of *KLF13* during ASVC early adipogenic differentiation (Additional file 1: Figure S1A, S2A).

On the basis of *KLF13* expression pattern, we predicted that KLF13 would promote porcine adipocyte differentiation and might play a role in the early adipogenic differentiation. To address the function of KLF13 in the early stage of porcine adipocyte differentiation, we established three different small interference RNA (siRNA) sequences against KLF13 and constructed KLF13 overexpression vector (Materials and methods). The mRNA and protein levels of KLF13 were significantly inhibited after three independent siRNAs treament (Fig. 1c). Expression vector for KLF13 significantly promoted mRNA and protein levels of KLF13 (Fig. 1d). Oil red-O staining on day 8 showed that three KLF13 siRNAs significantly diminished the accumulation of lipid droplets (Fig. 1e). Moreover, the mRNA levels of *PPARγ* and *aP2* were significantly suppressed by KLF13 siRNA1 and siRNA3 on day 8 compared to the expression level in the control siRNA group (Fig. 1f). As KLF13 siRNA3 showed the highest efficiency for inhibiting *KLF13* expression and ASVC adipogenic differentiation, the KLF13 siRNA3 was chosen for the subsequent experiments. We next examined whether overexpression of KLF13 might induce adipogneic differentiation in ASVC. As expected, KLF13 overexpression in ASVC promoted lipid accumulation and adipogenic gene expression, demonstrated by staining and gene expression patterns (Fig. 1g,h). Furthermore, we observed the effect of KLF13 knockdown in porcine MSVC and DFAT cells. The results showed that knockdown of KLF13 in porcine MSVC and DFAT cells suppressed adipogenic differentiation (Additional file 1: Figure S1B, S2B). In addition, Real-time PCR analysis showed that the mRNA expression levels of *PPARγ, aP2* and *Adiponectin* were significantly inhibited on day 8 of MSVC and DFAT cells adipogenic differentiation (Additional file 1: Figure S1C, S2C). Thus, the combined data from gain- and loss-of-function studies consistently demonstrate that KLF13 acts as an early promoter of porcine adipocyte differentiation.

### KLF13 promotes the major adipogenic factors *PPARγ* and *C/EBPα*

**Fig. 2:**
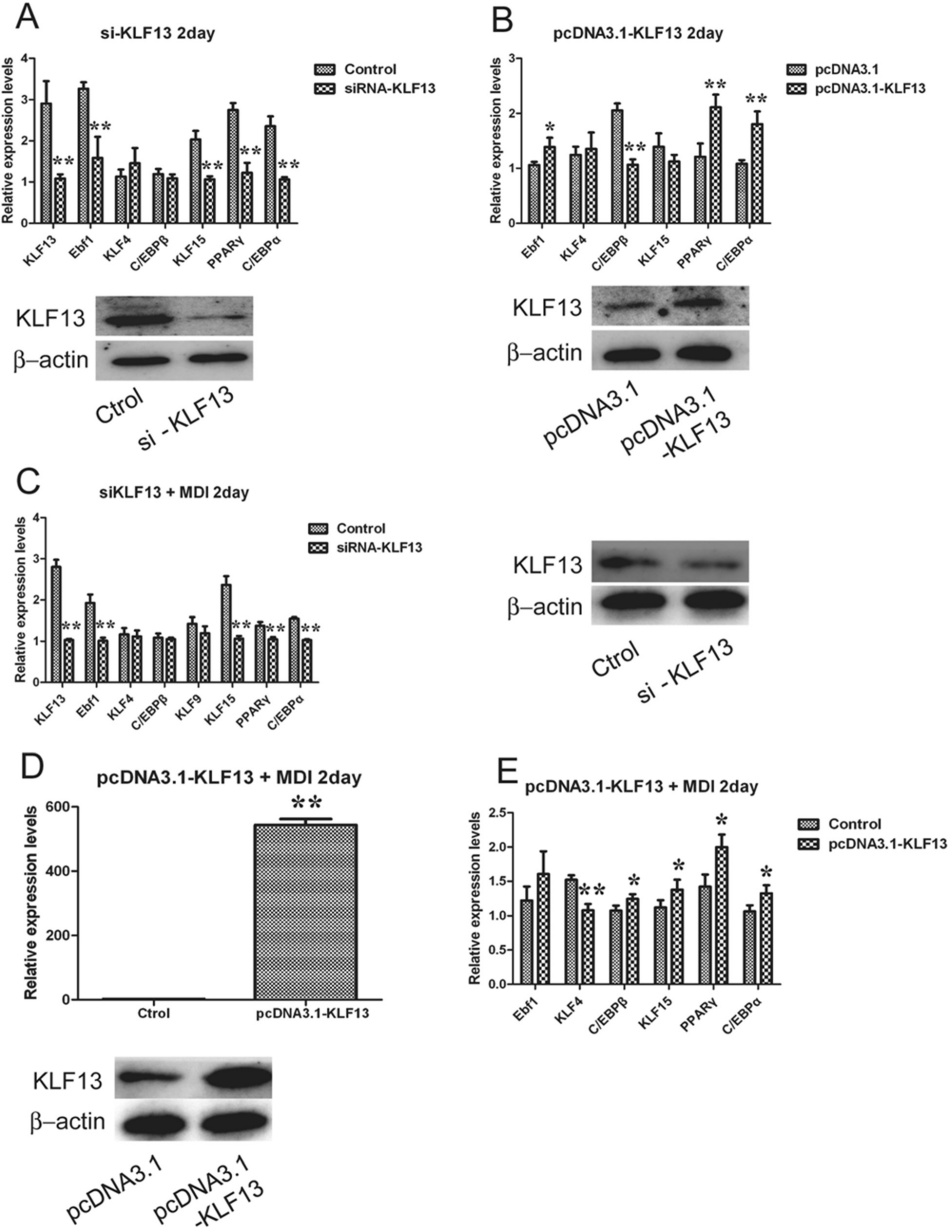
Effect of knockdown or overexpression of KLF13 on the expression of adipogenic factors. **a** After 2 days transfection of KLF13 siRNA, adipose SV cells were harvested. Real-time PCR was used to determine the mRNA expression of adipogenic factor genes *Ebf1, KLF4, C/EBPβ, KLF15, PPARγ* and *C/EBPα*. The protein level was determined by western blotting. Values are represented as mean ± SD. (n = 3). **b** After 2 days transfection of pcDNA3.1-KLF13, adipose SV cells were harvested. Real-time PCR was used to determine the mRNA expression of adipocyte differentiation-related genes *Ebf1, KLF4, C/EBPβ, KLF15, PPARγ* and *C/EBPα*. The protein level was determined by western blotting. Values are represented as mean ± SD. (n = 3). **c** After 1 days transfection of KLF13 siRNA, adipose SV cells were stimulated in adipogenic induction medium for 2 days. Real-time PCR was used to determine the mRNA expression of adipocyte differentiation-related genes *Ebf1, KLF4, C/EBPβ, KLF9, KLF15, PPARγ* and *C/EBPα*. The protein level was determined by western blotting. Values are represented as mean ± SD. (n = 3). **d** After 1 days transfection of pcDNA3.1-KLF13, adipose SV cells were stimulated in adipogenic induction medium for 2 days. Real-time PCR was used to determine the mRNA expression of *KLF13*. The protein level was determined by western blotting. Values are represented as mean ± SD. (n = 3). **e** After 1 days transfection of pcDNA3.1-KLF13, adipose SV cells were stimulated in adipogenic induction medium for 2 days. Real-time PCR was used to determine the mRNA expression of adipocyte differentiation-related genes *Ebf1, KLF4, C/EBPβ, KLF15, PPARγ* and *C/EBPα*. Values are represented as mean ± SD. (n = 3) *P < 0.05, **P < 0.01

KLF13 is a transcriptional regulator, we sought to identify downstream targets through both gain-of-function and loss-of-function studies under adipogenic or non-adipogenic induction (normal grown medium) conditions. In order to identify bona fide targets of KLF13, we focused on the known key adipogenic regulators that showed coordinate regulation between KLF13 overexpression and knockdown. In the context of normal grown medium, there are four transcription factor genes (*Ebf1, KLF15, PPARγ,* and *C/EBPα*) that were significantly diminished by KLF13 knockdown in ASVC (Fig. 2a), while three transcription factor genes (*Ebf1, PPARγ,* and *C/EBPα*) whose expression significantly increased upon treatment with KLF13 overexpression in ASVC, *C/EBPβ* was decreased (Fig. 2b). A total of three transcription factor genes (*Ebf1, PPARγ,* and *C/EBPα*) were coordinately regulated by KLF13 in ASVC under normal gown medium.

In the context of adipogenic induction, four transcription factor genes (*Ebf1, KLF15, PPARγ,* and *C/EBPα*) were significantly inhibited with the treatment of si-KLF13 in ASVC. In addition, knocking down KLF13 in porcine DFAT cells with siRNA also significantly inhibited the transcription factor genes (*KLF15, PPARγ,* and *C/EBPα*) expression (Additional file 1: Figure S3). Conversely, overexpressing KLF13 in ASVC under adipogenic induction condition (Fig. 2d) significantly promoted expression of the transcription factor genes (*C/EBPβ, KLF15, PPARγ,* and *C/EBPα*) (Fig. 2e). A total of three genes (*KLF15, PPARγ,* and *C/EBPα*) were coordinately regulated by KLF13 in ASVC under adipogenic induction condition. Taken together, these results demonstrate that *PPARγ* and *C/EBPα* are bona fide positive targets of KLF13 in the context of porcine ASVC adipogenic differentiation.

### KLF13 transactivates *PPARγ* through directly binding to the *PPARγ* promoter

**Fig. 3:**
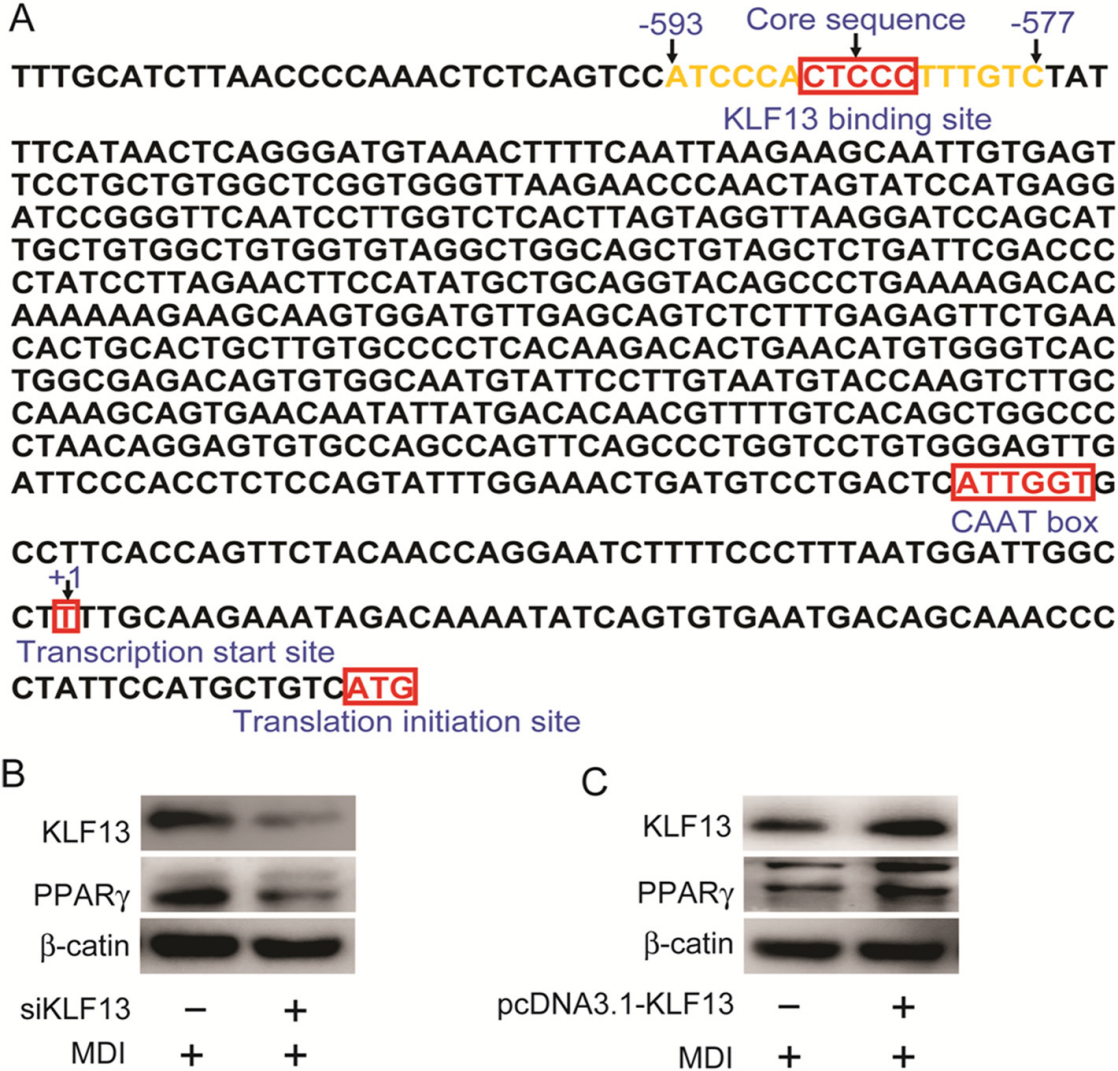
Effect of KLF13 on the expression of PPARγ during adipogenic differentiation of porcine ASVC. **a** Analysis of transcription factor site within the *PPARγ* promoter. Genomatix MatInspector was used to identify transcription factor site within the promoter of *PPARγ* from 2000 bp upstream of the translation initiation site. **b** Effect of KLF13 knockdown on the expression of PPARγ during adipogenic differentiation of porcine ASVC. After 1 days transfection of KLF13 siRNA, SV cells were stimulated in adipogenic induction medium for 3 days. Western-blot was used to determine the protein expression of PPARγ. **c** Effect of KLF13 overexpression on the expression of PPARγ during adipogenic differentiation of porcine ASVC. After 1 days transfection of pcDNA3.1-KLF13, SV cells were stimulated in adipogenic induction medium for 3 days. Western-blot was used to determine the protein expression of PPARγ. Values are represented as mean ± SD. (n = 3) *P < 0.05

The data shown above suggested that KLF13 would regulate *PPARγ* and *C/EBPα* gene expression. To assess whether KLF13 directly activates the *PPARγ* and *C/EBPα* gene, we next predicted the binding of KLF13 to the promoters of *PPARγ* and *C/EBPα* gene. Bioinformatics analysis showed that only *PPARγ* gene promoter had a potential binding site (CTCCC) for KLF13. No canonical TATA box was found in the *PPARγ* promoter region close to the transcription initiation site, whereas a CAAT box was found in the *PPARγ* promoter (Fig. 3a). Thus, we proposed *PPARγ* as potential KLF13 direct target during the process of porcine adipocyte differentiation. Real-time PCR analysis has shown that the *PPARγ* was coordinately regulated by KLF13 in ASVC (Fig. 2c,e). Furthermore, western blot analysis also confirmed that PPARγ expression was coordinately regulated by KLF13 in ASVC under adipogenic induction condition (Fig. 3b,c).

**Fig. 4:**
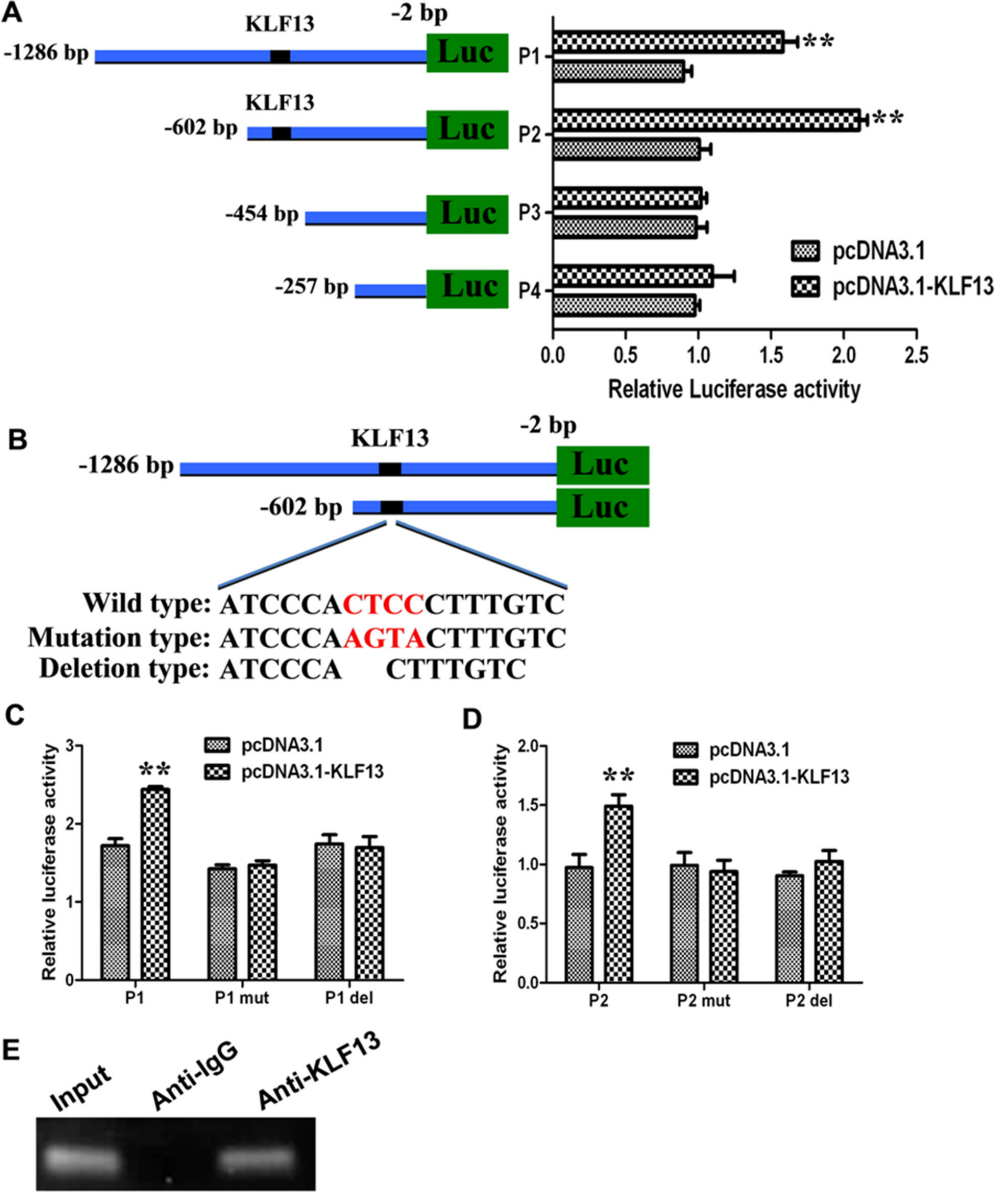
Activation of porcine *PPARγ* transcription by KLF13. **a** The truncation analysis of *PPARγ* promoter. The truncated *PPARγ* promoter reporters (P1 ~ P4) were respectively co-transfected with pcDNA3.1-KLF13 or empty vector into PK15 cells. The dual-luciferase activity was measured 36 h after transfection. Values are represented as mean ± SD. (n = 3). **b** Schematics structure of KLF13 binding site mutation and deletion. **c** Effect of KLF13 binding site mutation and deletion on P1 promoter activity. Wild (P1), mutation type (P1 mut) and deletion type (P1 del) of *PPARγ* promoter reporter were respectively co-transfected with pcDNA3.1-KLF13 into PK15 cells. The dual-luciferase activity was measured 36 h after transfection. Values are represented as mean ± SD. (n = 3). **d** Effect of KLF13 binding site mutation and deletion on P2 promoter activity. Wild (P2), mutation type (P2 mut) and deletion type (P2 del) of *PPARγ* promoter reporter were respectively co-transfected with pcDNA3.1-KLF13 into PK15 cells. The dual-luciferase activity was measured 36 h after transfection. Values are represented as mean ± SD. (n = 3). **e** ChIP assay of KLF13 binding to porcine *PPARγ* promoter. The porcine ASVC were induced with adipogenic medium for 3 days, the cells were harvested for ChIP experiment. KLF13 binding region was detected by PCR. **P < 0.01

Because we found that KLF13 plays an important role in PPARγ gene and protein expression, and bioinformatics analysis found a potential binding site for KLF13. We next examined whether KLF13 directly regulates transcription of *PPARγ* gene. To clarify which region is responsible for KLF13 transactivation, a series of deletions (P1, −1286 bp/-2 bp; P2, −602 bp/-62 bp; P3, −454 bp/-2 bp; P4, −257 bp/-2 bp) of a 1284 bp fragment of porcine *PPARγ* promoter were made and co-transfected into porcine PK15 cells with KLF13 overexpression plasmid, respectively (Fig. 4a). Among the four promoters, only P1 and P2 promoters contain the potential binding site for KLF13. The Fig. 4a showed that KLF13 significantly stimulated P1 and P2 promoter activity in reporter assays, but these activation were almost completely abolished when the P3 and P4 promoters were used. These results indicated that KLF13 plays a critical role in porcine *PPARγ* promoter activity and the direct binding site exists between −602 and −454 bp in the porcine *PPARγ* promoter.

Sequence analysis of the porcine *PPARγ* promoter has revealed a potential binding core sequence (CTCCC) for KLF13. Of these, Site-specific mutation or deletion in both P1 and P2 promoters were performed (Fig. 4b) to analyze the activity of these mutated and deleted porcine *PPARγ* promoters by luciferase report assay. The results showed that either mutant significantly reduced the activity of P1 and P2 promoters induced by KLF13 in porcine PK15 cells (Fig. 4c,d). These results suggested that the potential binding sequence be functionally important. To assess whether KLF13 could bind specifically to the *PPARγ* promoter during porcine adipocyte differentiation, we analyzed the binding activity of KLF13 to the endogenous PPARγ promoter in porcine ASVC by ChIP assays. ChIP results showed the significant KLF13 binding to the *PPARγ* promoter was detected at day 3 after MDI induction (Fig. 4e). Taken together, the results indicate that KLF13 could directly bind to the promoter region of *PPARγ* gene during the porcine adipocyte differentiation.

### KLF13 is not necessary for adipocyte differentiation in mouse 3 T3-L1 preadipocytes

**Fig. 5:**
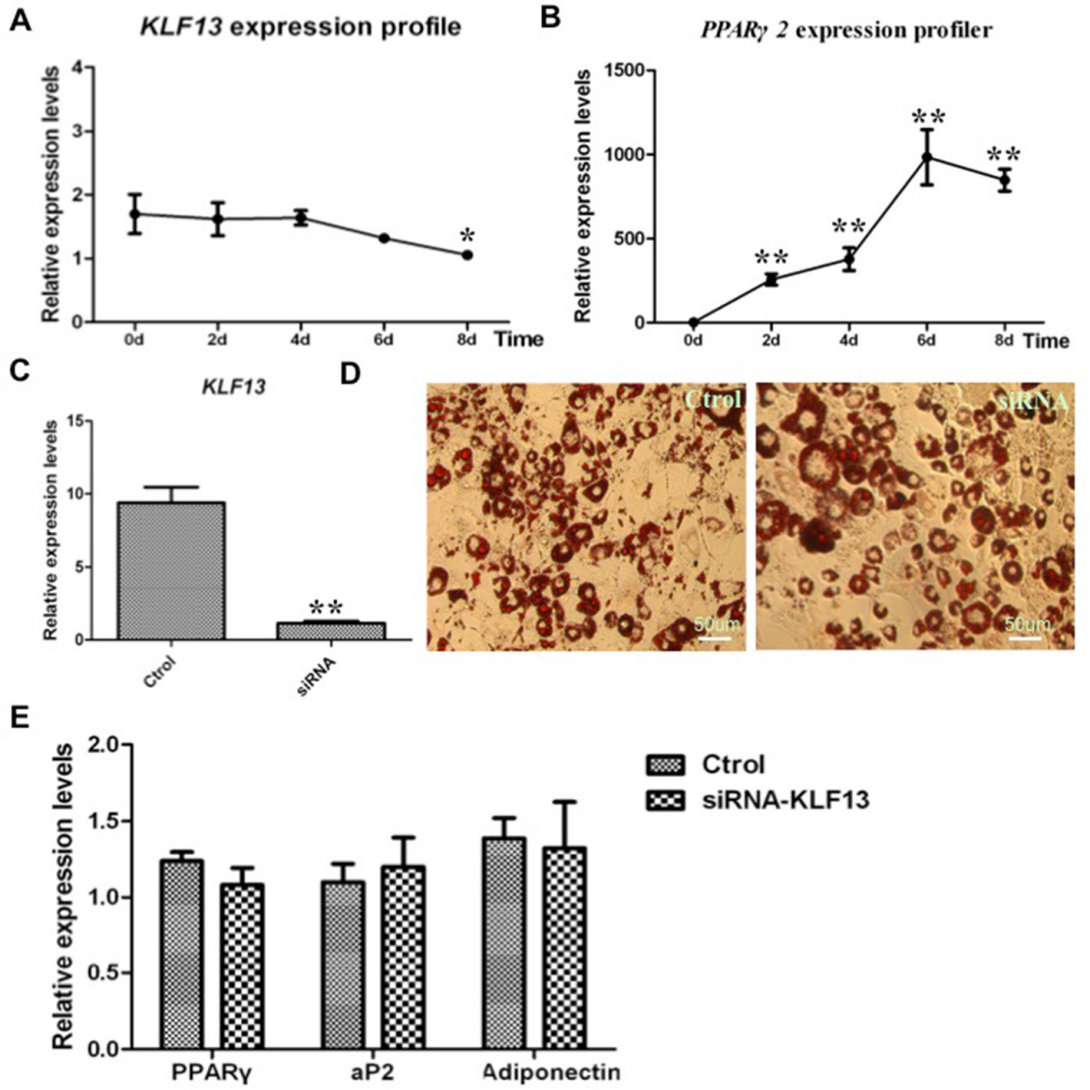
Expression and function of KLF13 in mouse 3 T3-L1 preadipocytes. **a** The mRNA expression of *KLF13* in mouse 3 T3-L1 preadipocytes during adipocyte differentiation. The mRNA level was determined by real-time PCR and normalized to β-actin mRNA. The numbers indicate the time points of differentiation induction. Results are expressed as means ± SD. (n = 3). **b** The mRNA expression of *PPARγ2* in mouse 3 T3-L1 preadipocytes during adipocyte differentiation. The mRNA level was determined by real-time PCR and normalized to β-actin mRNA. The numbers indicate the time points of differentiation induction. Results are expressed as means ± SD. (n = 3). **c** The KLF13 mRNA level in ASVC with the treatment of siRNA. siRNA was the siRNA sequences against mouse KLF13 (see Materials and Methods for details). Ctrol indicates control siRNA sequence. Endogenous KLF13 mRNA was determined by real-time PCR in pre-induction. Results are expressed as means ± SD. (n = 3). **d** Effect of knockdown KLF13 on adipogenic differentiation of 3 T3-L1 cells. 3 T3-L1 preadipocytes were treated with KLF13 siRNA at about 70 % confluence. After 24 h, the cells were induced to adipogenic differentiation. On day 8, the cell monolayer was stained with Oil-red O. **e** The mRNA expression of *PPARγ*, *aP2* and *Adiponectin* in KLF13-knockdown 3 T3-L1 cells were detected by real-time PCR on day 8 after adipogenic induction. Results are expressed as means ± SD. (n = 3) *P < 0.05, **P < 0.01

To assess whether KLF13 was related to mouse adipocyte differentiation, we analyzed the expression pattern of *KLF13* during adipocyte differentiation of 3 T3-L1 preadipocytes. Real-time PCR analysis showed that *KLF13* mRNA did not significantly changed during the first 6 days of 3 T3-L1 preadipocytes differentiaiton process (Fig. 5a). As expected, the mRNA level of adipogenesis-specific marker gene such as *PPARγ2* increased during the process of 3 T3-L1 preadipocytes differentiation (Fig. 5b). We next evaluated the role of KLF13 in 3 T3-L1 preadipocytes differentiation using siRNA for mouse KLF13 to knock down its expression. The siRNA sequence for mouse KLF13 was effective in knocking down *KLF13* expression (Fig. 5c). Oil red-O staining on day 8 revealed that KLF13 siRNA did not effectively diminished the accumulation of lipid droplets compared to the control group (Fig. 5d). Moreover, the expression levels of *PPARγ2, aP2* and *Adiponectin* were also not significantly changed by KLF13 siRNA on day 8 compared to expression levels in the control group (Fig. 5e).

**Fig. 6:**
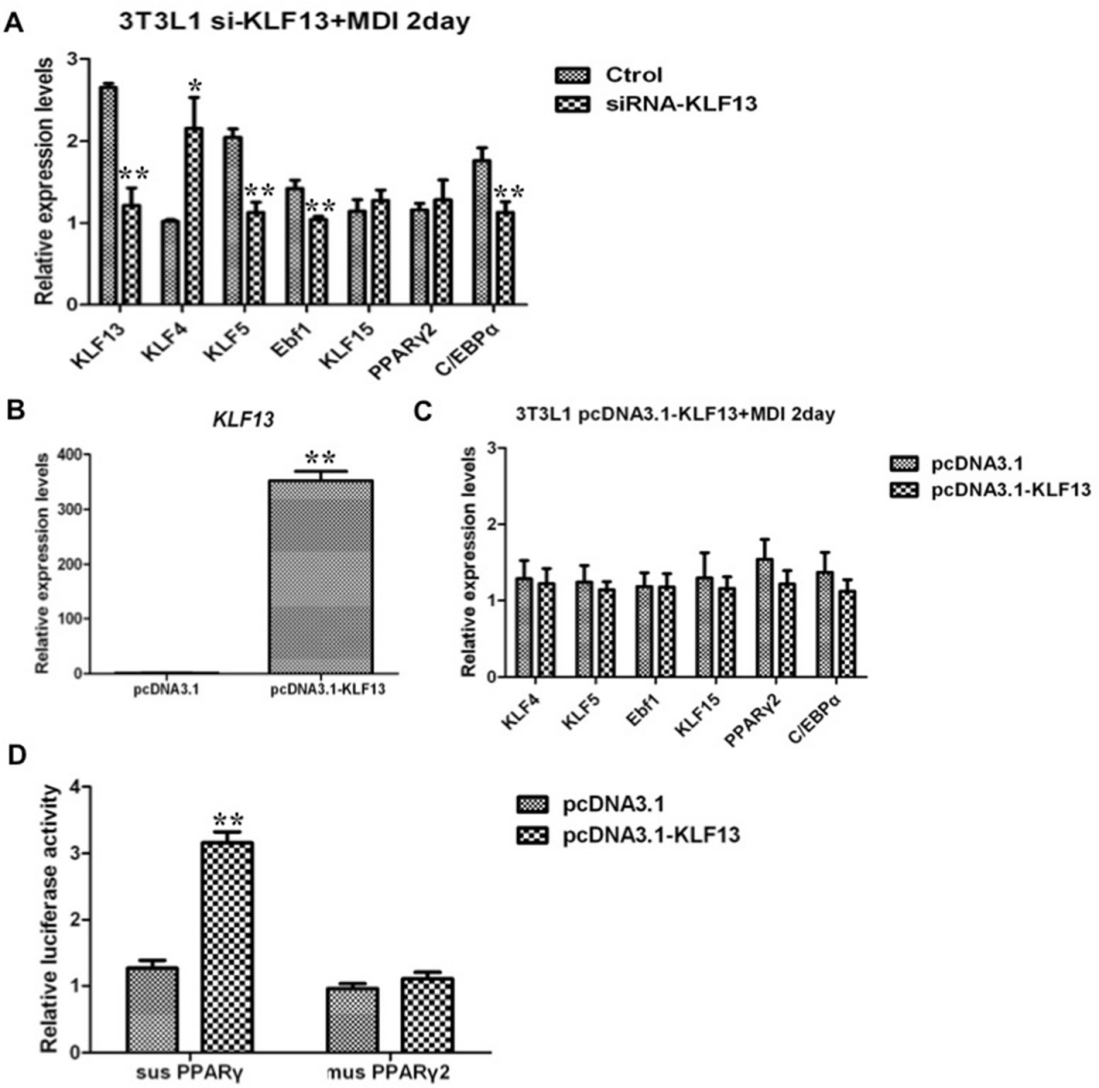
Transcriptional regulation of mouse key adipogenic factors by KLF13. **a** Effect of knockdown KLF13 on the expression of adipogenic factor genes during adipogenic differentiation of 3 T3-L1 cells. After 1 days transfection of KLF13 siRNA, adipose 3 T3-L1 were stimulated in adipogenic induction medium for 2 days. Real-time PCR was used to determine the mRNA expression of *KLF13, KLF4, KLF5, Ebf1, KLF15, PPARγ2* and *C/EBPα*. Values are represented as mean ± SD. (n = 3). **b** The *KLF13* mRNA level in overexpression of *KLF13* during adipogenic differentiation of 3 T3-L1 cells. After 1 days transfection of pcDNA3.1-KLF13, 3 T3-L1 cells were stimulated in adipogenic induction medium for 2 days. Real-time PCR was used to determine the mRNA expression of *KLF13.* Values are represented as mean ± SD. (n = 3). **c** Effect of overexpression of *KLF13* on the expression of adipogenic factor genes during adipogenic differentiation of 3 T3-L1 cells. After 1 days transfection of pcDNA3.1-KLF13, 3 T3-L1 cells were stimulated in adipogenic induction medium for 2 days. Real-time PCR was used to determine the mRNA expression of *KLF4, KLF5, Ebf1, KLF15, PPARγ2* and *C/EBPα*. Values are represented as mean ± SD. (n = 3). **d** Effect of overexpression KLF13 on porcine *PPARγ* and mouse *PPARγ2* promoters activity. Porcine *PPARγ* and mouse *PPARγ2* promoter reporter were respectively co-transfected with pcDNA3.1-KLF13 into 3 T3-L1 cells. The dual-luciferase activity was measured 36 h after transfection. Values are represented as mean ± SD. (n = 3) *P < 0.05, **P < 0.01

Furthermore, we examined whether KLF13 regulated expression of the known adipogenic-related transcription factor genes in 3 T3-L1 preadipocytes with the MDI induction. The results showed that three transcription factor genes (*KLF5, Ebf1,* and *C/EBPα*) were significantly diminished by KLF13 knockdown in 3 T3-L1 preadipocytes in the context of MDI induction (Fig. 6a). However, overexpression of KLF13 in preadipocytes did not affect the expression of these transcription factor genes (Fig. 6c). Taken together, the combined data from gain- and loss-of-function studies demonstrate that the known adipogenic-related transcription factor genes, in particular for *PPARγ2,* were not coordinately regulated by KLF13 in 3 T3-L1 preadipocytes, suggesting that there be species-specific differences in regulating expression of porcine *PPARγ* gene and mouse *PPARγ2* gene by KLF13. This was confirmed through luciferase report assay. The results showed that KLF13 could significantly activate the porcine *PPARγ* promoter, whereas no such effect was detected on the promoter of mouse *PPARγ2* (Fig. 6d).

## Discussion

### KLF13 affects porcine adipocyte differentiaiton

During the last thirty years, investigators have embarked on a detailed and systematic endeavor to define the transcriptional events regualting preadipocyte differentiation. The differentiaiton of preadipocytes into adipocytes is regualted by an elaborate network of transcription factors that coordinate expression of hundreds of proteins responsible for establishing the mature fat-cell phenotype [4]. In recent years, many members of KLFs have been identified to be involved in adipogenesis [10]. So far, a total of nine KLF members have been identified to regulate adipocyte differentiaiton, of which KLF4, KLF5, KLF6, KLF8, KLF9, and KLF15 promote adipocyte differentiaiton whereas KLF2, KLF3, and KLF7 inhibit adipocyte differentiaiton [10]. Thus, the KLFs emerging during adipocyte differentiaiton show important roles in regulating this process.

KLF13 as a member of KLFs family previously identified to be important for various physiological processes. For example, KLF13 is an important component of the transcription network required for heart development and suggests that KLF13 be a GATA-4 modifier, by analogy to other GATA-4 collaborators, mutations in KLF13 may be causative for congenital human heart disease [11]. In addition, KLF13 is required for orchestrating the induction of RANTES (regulated upon activation normal T cell expressed and secreted) expression in T lymphocytes [13]. Furthermore, KLF13 may function in regulation of cholesterol homeostasis. KLF13 binds to three TC-predominant DNA sequences in the low density lipoprotein receptor (*LDLR*) gene in vitro and to the proximal *LDLR* promoter in vivo, represses *LDLR* promoter activity [12]. However, to date, there has been no evidence regarding the functional role of KLF13 in adipocyte differentiaiton. In our previous study, porcine adipose SV cells were isolated, and then induced to differentiaiton into adipocytes *in vitro*. RNA-Seq was used to screen differentially expressed genes during the adipocyte differentiaion of porcine adipose SV cells on days 0, 2, and 4. We found that the expression of *KLF13* was significantly up-regulated during porcine adipose SV cells adipogenic differentiation [14]. This result suggested that KLF13 might be a key intermediate component of the transcription factor network regulating porcine adipocyte differentiation.

In this study, we used three types of porcine primary preadipocytes (ASVC, MSVC and DFAT cells) to confirm that *KLF13* expression was induced at the early stage of porcine adipocyte differentiation. Of these, we speculated that KLF13 might be involved in porcine adipocyte differentiaiton. We next investigeted the role of KLF13 in porcine ASVC adipogenic differentiaiton. siRNA-mediated knockdown of endogeous KLF13 expression in ASVC lead to the inhibition of lipid accumulation and expression of adipogenic markers. Consistently, overexpression it could promote ASVC adipogenic differentiaiton. In addition, we used MSVC and DFAT cell models to also confirm that downregulation of KLF13 by siRNA impaired adipocyte differentiaiton. These results suggest that KLF13 is required and sufficient to perform a full program of porcine adipocyte differentiaiton.

### KLF13 directly transactivates poricne *PPARγ*

Adipocyte differentiaiton is controlled by a tightly regulated transcriptional cascade where the transcription factors activate or repress the expression of each other in a sequential manner. PPARγ as a key regulator of adipocyte differentiation plays a center role in this transcriptional cascade. This is because PPARγ is not only crucial for inducing expression of the gene program that leads to the mature adipocyte phenotype [4, 6, 15], but is also required for mediating the early adipogenic factors regulating adipocyte differentiation [3]. Adipocyte differentiation is the result of various transcription factors that function in the complex transcriptional cascade. The adipogenic cascade can be divided into at least two waves of transcription factors that drive the adipogenic program. The first wave is initiated by adipogenic stimuli that activate several early adipogenic factors. And these transcription factors in turn induce expression of the second wave of transcription factors, of which PPARγ and C/EBPα are the most important [3]. The most early pro-adipogenic transcription factors seem to function at least in part by activating PPARγ expression or activity [6, 16]. By contrast, anti-adipogenic GATA factors function in part by repressing PPARγ expression [17]. Different early adipogenic factors can function through the same transcription factor (PPARγ) to regulate adipocyte differentiation.

In the present study, we found that KLF13 is an early pro-adipogenic factor. In order to investigate the regulatory manner of KLF13 and identify its targets, we performed the gain-of function and loss-of function experiments in both normal grown and adipogenic induction contexts to enhance our ability to identify bona fide targets of KLF13, focusing on genes that showed coordinate regulation between KLF13 overexpression and knockdown. The combined data demonstrate that *PPARγ* and *C/EBPα* are bona fide positive targets of KLF13 in the process of porcine ASVC adipogenic differentiation. Subsequently, we examined whether the promoters of porcine *PPARγ* and *C/EBPα* had the binding sites for KLF13. As we expected, there is a potential binding site (CTCCC) for KLF13 to bind to the porcine *PPARγ* promoter (Fig. 3a). Thus, we proposed *PPARγ* as potential KLF13 direct target during porcine adipocyte differentiation. Promoter truncation, mutation and deletion assays all proved that KLF13 could transactivate the porcine *PPARγ* promoter. In addition, the results of ChIP assay demonstrated that KLF13 could bind specifically to the *PPARγ* promoter during porcine adipocyte differentiation. Taken together, these results indicate that PPARγ is the direct target of KLF13 during porcine adipocyte differentiation.

KLF13 as a member of KLFs family contains three highly conversed classical C2H2 zinc fingers [8]. These fingers are located at the carboxyl terminus and enable KLFs to specifically bind to GC-rich sequences and related GT and CACCC boxes in regulatory regions of target genes [18]. In present study, we found that KLF13 binding site on the *PPARγ* promoter is a CACCC box (Fig. 3a) and activate the porcine *PPARγ* promoter. However, individual members of KLF family can function as activators or repressors depending on which promoter they bind and the coregulators with which they interact in the non-DNA-binding regions [19]. In previous studies, KLF13 has been found to can recruit coactivators/corepressors such as CBP/p300, PCAF and ctBP2 [19–21]. Whether recruitment of co-regulators are involved in the regulation of porcine adipocyte differentiation by KLF13 needs to be further investigated.

### Species-specific regulation of porcine and mouse adipocyte differentiation by KLF13

Recently, a series of experiments demonstrated that nine KLF members paly vital roles in adipocyte differentiation using a variety of mouse preadipocyte cell culture models [10, 22]. But KLF13 is not included in these nine KLF members. However, the present study demonstrated that KLF13 function has a strong impact on porcine adipocyte differentiation. To test whether KLF13 could regulate mouse adipocyte differentiation, we firstly analyzed the expression pattern of *KLF13* during adipocyte differentiation of 3 T3-L1 preadipocytes. Subsequently, we evaluated the role of KLF13 in 3 T3-L1 preadipocytes differentiation using loss-of-function experiment. The data suggested that KLF13 did not influence mouse adipocyte differentiation. Furthermore, we examined the effect of KLF13 on the expression of the known adipogenic factor genes. The results showed that overexpression and knockdown of KLF13 did not influence the mouse *PPARγ2* expression. It is noteworthy that the above results have proved that KLF13 promoted porcine adipocyte differentiation through transactivating *PPARγ*. Thus, we speculated that KLF13 regulating porcine and mouse adipocyte differentiation may exist species-specific mechanisms. This species-specific event is likely due to the difference in regulating porcine *PPARγ* and mouse *PPARγ2* by KLF13. Bioinformatics analysis found a potential binding site for KLF13 in porcine *PPARγ* promoter, while no binding site was obtained for KLF13 in mouse *PPARγ2* promoter using same bioinformatics analysis method. Subsequently, we compared the DNA sequences of porcine *PPARγ2* promoter (2000 bp), human *PPARγ2* promoter (2000 bp) and mouse *PPARγ2* promoter (2000 bp) and found that their homology is about 50 %. Furthermore, the core sequence (CTCCC) of KLF13 binding is specificity on the promoter of porcine *PPARγ2* (Additional file 1: Figure S4). These results imply that the transcriptional regulations of *PPARγ* may exist sizable divergence between porcine and mouse. Finally, the present study confirmed that KLF13 could effectively activate porcine *PPARγ* promoter in mouse 3 T3-L1 cells whereas no such effect was detected on mouse *PPARγ2* promoter (Fig. 6d). These findings could perhaps explain the species-specific difference in regulation of porcine and mouse adipocyte differentiation by KLF13. These results further suggest that KLF13 may be a specific transcription regulatory factor of porcine adipocyte differentiation. KLF13 may also be associated with pig meat quality traits, especially the development of intramuscular fat. We demonstrated that there was good implication for potential application in animal nutrition or genetic selection may provide effective ways to improve health and management of animals.

In summary, we reported the identification of KLF13 as an essential player in porcine adipocyte differentiation. KLF13 is a key pro-adipogenic factor through regulating *PPARγ* transactivation at the early stage of porcine adipocyte differentiation, whereas no such effect was detected during mouse adipocyte differentiation. The present findings provide interesting evidence for the species-specific regulation of adipocyte differentiation by KLF13, which would provide inspiration for other KLF members in regulating adipocyte differentiation between different species.

## Materials and methods

### Cell isolation and cuture

Large white pigs (provided by Jingpin Pig farm of National Engineering Research Center on Farm Animals) were sacrificed at 5 days of age by intraperitoneal injection of pentobarbital sodium (50 mg/kg body weight) followed by exsanguination. Subcutaneous adipose tissue (SAT) and *longissimus dorsi* muscle (LM) were aseptically isolated and finely minced after removing all visible connective tissues. Porcine subcutaneous stromal vascular cells (ASVC) and intramuscular stromal vascular cells (MSVC) were obtained based on previously reported methods with some modifications [23–25]. SAT and LM tissues were treated with digestion solution comprising 0.1 % type-I and 0.2 % type-II collagenase (Sigma), respectively, for 2 h at 37 °C, followed by centrifugation of the digestion mixture at 1000 × g for 8 min. Afterwards, the resulting mixture was filtered through 100 and 40 μm mesh filters and centrifuged for another 8 min at 1000 × g to obtain SV cell pellets. These cell pellets were plated in proliferation medium comprising 90 % DMEM and 10 % fetal bovine serum (FBS), both from Gibco (Grand Island, NY, USA). All Animal were maintained and used in accordance with the guidelines of the Institutional Animal Care and Use Committees at the Huazhong Agricultural University.

Primary adipocytes were isolated based on the previously reported methods with modifications [25]. Mature adipocytes were isolated from porcine subcutaneous adipose tissue. To isolate mature adipocytes, the floating primary mature adipocytes in the top layer after filtration and centrifugation at 220 g for 5 min were collected. After three wash with phosphate-buffered saline (PBS), cells were placed in 25 cm^2^ culture flasks (Corning Inc., NY, USA) filled completely with medium supplemented with 15 % FBS and were incubated at 37 °C in 5 % CO2. Mature adipocytes floated up and adhered to the top inner ceiling surface of the flasks. Approximately 1 week later, the cells were firmly attached to the ceiling and changed into fibroblast-like dedifferentiated fat cells (DFAT cells) with no visible fat droplets. Then, the medium was removed and changed into DMEM medium supplemented with 15 % FBS, and the flasks were inverted so that DFAT cells were on the bottom. The medium was changed every 4 days until DFAT cells reached to confluence [26].

ASVC, MSVC, 3 T3-L1 preadipocytes and PK15 cells were maintained in DMEM with 10 % FBS. DFAT cells were maintained in DMEM with 15 % FBS.

### Differentiation induction *in vitro* and Oil-red-O staining

For adipogenic differentiation, ASVC, MSVC, DFAT cells and 3 T3-L1 preadipocytes were induced with a cocktail of dexamethasone (1 μM), 3-isobutyl-1-methyxanthine (0.5 mM), and insulin (10 μg/mL) (DMI) beginning 2 d after confluence was reached (day 0). After 2 d, media was changed to DMEM/10 % FBS plus insulin (10 μg/mL) for an additional 2 d and thereafter maintained in DMEM containing 10 % FBS.

Oil red-O staining was performed on adipogenic differentiation day 8 of preadipocytes. Briefly, cells were washed three times in PBS and then fixed in 4 % (v/v) formaldehyde for 10 min. Subsequently, the fixed cells were rapidly washed with PBS. In the end, 0.5 % oil red-O was added to the cells for 15 min to visualize lipid droplets stained red.

### KLF13 knockdown and overexpression

KLF13 and control small interfering RNAs (siRNAs) were used to knockdown experiments. Based on the information of the porcine KLF13 cDNA sequence (GenBank accession no. NM_001011505.1), three specific siRNAs for porcine KLF13 and universal negative control dicer substrate duplex were synthesized and purchased from RiboBio Co. Ltd (Guangzhou, China). The targeted sequences of porcine KLF13 were siRNA1, 5′-GACCTTAACCAGCAAGCAC-3′; siRNA2, 5′-GCACCTGAGAACTCACACA -3′ and siRNA3, 5′-GGCAGGACTGCAACAAG AA-3′. Cells were transfected with control or KLF13-specific siRNA in OPTI-MEM medium using Lipofectamine RNAiMAX (Invitrogen), according to the manufacturer’s protocol. The next day, the medium was replaced with fresh DMEM containing 10 % FBS and the cells were incubated for 24 h before the induction of adipogenic differentiation. Total RNA and protein extracts were prepared from the cells at the indicated time points, and real-time PCR and western-blot analyses were performed. Oil red-O staining of KLF13 knockdown was performed at day 8.

KLF13 overexpression vector (pcDNA3.1-KLF13) was generated by inserting the whole open reading frame (ORF) of porcine KLF13 into pcDNA3.1 (Invitrogen). The whole ORF of KLF13 was PCR-amplified from a pig adipose cDNA using the specific primers (forward: 5′-CCC**AAGCTT**ATGGCAGCCGCCGCCTATG-3′ and reverse: 5′-CG**GAATTC**TCAGGGCGAGCTTGCCGG-3′). The purified PCR product was cloned into pcDNA3.1 (Invitrogen). Cells were transfected with KLF13 overexpression vector by Lipofectamine 2000 (Invitrogen), according to the manufacturer’s protocol.

### RNA extraction and real-time PCR

Total RNA was extracted from cells or tissues using Trizol reagent (Invitrogen) according to the manufacturer’s instructions. cDNA was reverse-transcribed from 2.5 ug of RNA by using the first strand cDNA synthesis kit (Toyobo). Real-time PCR was performed with iTaq Universal SYBR Green Supermix (Bio-Rad) using the CFX96 system (Bio-Rad). The relative amount of mRNA normalized to β-actin was calculated by using the delta-delta method [27]. Results were analyzed using a one-way analysis of variance (ANOVA) statistical test. Primer pairs for specific target genes were designed as listed in Additional file 1: Table S1. All real-time PCR experiments were carried out on three biological replicates with three technical replicates for every sample.

### Western blot analysis

For protein analysis, cells were washed with cold PBS and then lysed directly in 60 mM Tris–HCl (pH 6.8) containing 1 % (w/v) SDS. The lysate was centrifuged for 5 min at 13,000 rpm, and supernatants were quantified using a BCA protein assay and then frozen until further analysis. For western blots, cell extracts were subjected to SDS-PAGE separation and then transferred onto a polyvinylidene difluoride (PVDF) membrane. Blots were blocks with 5 % skim milk and incubation with the primary antibody overnight at 4 °C, followed by incubation with the secondary antibody for 1 h at room temperature and measured with the enhanced chemiluminescence (ECL) detection kit (Thermo Fisher Scientific). Protein expression was normalized by β-actin. Primary antibodies used for blotting were anti-KLF13 (AT263a, Abnova), anti-PPARγ (2435, Cell Signaling Technology), and anti-β-actin (sc-47778, Santa Cruz). Anti-mouse or rabbit IgG-HRP (Santa Cruz) were used to detect primary antibodies.

### Promoter cloning and luciferase reporter assay

The porcine PPARγ promoter (−1270 bp to +13 bp from the putative transcription start site) was amplified by PCR using sense 5′ TC**GAGCTC**CACAATTCCTCGCC AA 3′ and the antisense primers 5′ CCG**CTCGAG**GCCAATCCATTAAAGG 3′. The underlined areas indicate the sequences added for the restriction sites of SacI and XhoI, respectively. The DNA fagment was cloned into pGL3-basic plasmid in the upstream of the luciferase reporter gene (Promega, Madison, WI). Other deletion fragments were generated by PCR using this plasmid DNA as a template (see Additional file 1: Table S2 for PCR primer sequences). The PCR products were subcloned into the pGL3-basic plasmid in the upstream of the luciferase reporter gene (Promega, Madison, WI). All the sequences of the cloned promoter region were confirmed by DNA sequencing.

Site-directed mutagenesis or deletion to inactivate the KLF13-binding sites at positions −571 to −567 of porcine PPARγ promoter were carried out within the p-1270/+13 construct and p-586/+13 construct. The PCR primers were as follow: mutant KLF13 site forward, 5′-CTCAGTCCATCCCAAGTACTTTGTCTATTTCA- 3′, mutant KLF13 site reverse, 5′-TGAAATAGACAAAGTACTTGGGATGGAC TGAG-3′; deletion KLF13 site forward, 5′-CTCTCAGTCCATCCCACTTTGTC TATTTCATA-3′, deletion KLF13 site reverse, 5′-TATGAAATAGACAAAGTGGG ATGGACTGAGAG-3′. PCR amplification was performing using 50 ng template DNA and 22 cycles of 94 °C for 30s, 55 °C for 30s and 72 °C for 30s/kb. PCR products were digested with *DpnI* (Takara) for 2 h at 37 °C. All mutations and deletions were confirmed by DNA sequencing.

The mouse PPARγ2 promoter region spanning −2501 to +51 bp was amplified by PCR using sense 5′ **GGTACC**CATAGGGCAAAAGAAGGGCGTTA 3′ and the antisense primers 5′ **AAGCTT**AGAGATTTGCTGTAATTCACACTGG 3′. The underlined areas indicate the sequences added for the restriction sites of KpnI and HindIII, respectively. The DNA fagment was cloned into the pGL3 basic luciferase reporter to construct pGL3-mG2p2500 (Promega, Madison, WI). The sequence of the cloned promoter region was confirmed by DNA sequencing. The sequences of the porcine PPARγ promoter and mouse PPARγ2 promoter were analyzed for the consensus transcription factor binding sites using the MatInspector program (Genomatix Software).

For luciferase assays of the promoter constructs, promoter constructs and porcine KLF13 overexpression vector were co-transfected in porcine PK15 cells and 3 T3-L1 preadipocytes using Lipofectamine 2000 (Invitrogen), respectively. Luciferase activity was determined using the dual luciferase reporter assay system (Promega). Normalized luciferase activity was expressed as the ratio of firefly luciferase activity to *Renilla* luciferase of each sample. Transfections were performed in triplicate for each independent experiment.

### Chromatin immunoprecipitation (ChIP) Assay

ChIP assays were performed according to the ChIP Assay Kit (Beyotime) manufacturer instructions. Briefly, ASVC were fixed by adding formaldehyde to a final concentration of 1 % and incubated at 37 °C for 10 min to allow cross-linking of endogenous proteins and DNA. Following three times of wash with cold PBS supplemented with 1 mM PMSF, the cells were resuspended using a buffer containing 1 % SDS and 1 mM PMSF, and lysed by sonication using a sonicator (Scientz-IID, China). After centrifugation, the supernatant was collected and the chromatin in the supernatant was immunoprecipitated with antibodies anti-KLF13 (sc-9605, Santa Cruz) or normal mouse IgG control (Santa Cruz). Input control and the DNA obtained from the immunoprecipitation were amplified by PCR using primers specific to the porcine PPARγ promoter containing KLF13 binding site using the following primers: sense 5′-AACAGGACCTCATTGCTTATC-3′, antisense, 5′-CCACAGCAGGAAC TCACAAT-3′.

### Statistical analysis

All results were presented as mean ± SD from three independent experiments. Statistical comparisons of groups were made using one-way ANOVA. The *P* value of less than 0.05 was deemed statistically significant (*P < 0.05 and **P < 0.01).

## Additional file

Additional file 1: Table S1. Primers for real-time PCR assay. **Table S2.** Primers for the promoter truncation assay. **Figure S1.** Expression and function of KLF13 in porcine MASV. **Figure S2.** Expression and function of KLF13 in porcine DFAT cells. **Figure S3.** Effect of knockdown KLF13 on the expression of adipogenic factors during adipogenic differentiation of porcine DFAT cells. **Figure S4.** Sequence of the promoter of the pig, human and mouse PPARγ2 genes.

## Abbreviations

KLF: Kruppel-like factors
BTEB3: Basic transcription element-binding protein-3
SAT: Subcutaneous adipose tissue
LM: *Longissimus dorsi* muscle
SVC: Stromal vascular cells
FBS: Fetal bovine serum
DFAT: Dedifferentiated fat
siRNA: Small interfering RNAs
PVDF: Polyvinylidene difluoride
ECL: Enhanced chemiluminescence
ChIP: Chromatin immunoprecipitation
LDLR: Low density lipoprotein receptor
