# Supplementary material for: KLF13 promotes porcine adipocyte differentiation through PPARγ activation
Source: Cell Biosci. 2015 Jun 10;5:28. doi: 10.1186/s13578-015-0016-z (PMC4469396; doi:10.1186/s13578-015-0016-z)
Supplement: Additional file 1: Table S1. — Primers for real-time PCR assay. Table S2. Primers for the promoter truncation assay. Figure S1. Expression and function of KLF13 in porcine MASV. Figure S2. Expression and function of KLF13 in porcine DFAT cells. Figure S3. Effect of knockdown KLF13 on the expression of adipogenic factors during adipogenic differentiation of porcine DFAT cells. Figure S4. Sequence of the promoter of the pig, human and mouse PPARγ2 genes. [file 13578_2015_16_MOESM1_ESM.doc]

Table S1 Primers for real-time PCR assay

| Gene Symbol | Primers (Sense/Anti-sense 5’-3’) | Product length (bp) | Ta (℃) |
| --- | --- | --- | --- |
| *Sus KLF13* | CGGGCTGTGAGAAAGTTTACGG | 186 | 60 |
|  | ATGAAGCGTTTGTCGCAGATGG |  |  |
| *Sus PPARγ* | AGAGTATGCCAAGAACATCC | 261 | 56 |
|  | AGGTCGCTGTCATCTAATTC |  |  |
| *Sus aP2* | AAGTCAAGAGCACCATAACC | 119 | 56 |
|  | GATACATTCCACCACCAACT |  |  |
| *Sus Adiponectin* | TTGAAGGATGTGAAGGTCAG | 229 | 56 |
|  | CAATGTTGTGGTAGAGAAGG |  |  |
| *Sus Ebf1* | CCAACTTCTTCCACTTCGT | 177 | 59 |
|  | GCTCCGTCCTTATTCCATT |  |  |
| *Sus KLF4* | CCTCTCCAACTCACTGTCT | 377 | 59 |
|  | GCGATGCCTTCAACACAA |  |  |
| *Sus C/EBPβ* | GTCCAAACCAACCGCACAT | 262 | 58 |
|  | GAAACAACCCCGTAGGAACAT |  |  |
| *Sus KLF9* | GGGACACCTGGAAGGATTATT | 334 | 58 |
|  | GCTTTGAGATGGGAGGATTTT |  |  |
| *Sus KLF15* | GCATGGTGGACCACTTGCTT | 184 | 60 |
|  | CAAAGGGCTTGCGAGTCAGG |  |  |
| *Sus C/EBPα* | CTCACCGCTCCGATTCCTAC | 233 | 59 |
|  | AAGCCCCAAGTCCCTGTGTT |  |  |
| *Sus β-actin* | CCAGGTCATCACCATCGG | 158 | 60 |
|  | CCGTGTTGGCGTAGAGGT |  |  |
| *Mus KLF13* | TATGTGGACCACTTTGCCGCC | 203 | 60 |
|  | TGCTGGTTGAGGTCCGCTAGGAT |  |  |
| *Mus PPARγ2* | TGGGTGAAACTCTGGGAGATTC | 150 | 60 |
|  | AGAGGTCCACAGAGCTGATTCC |  |  |
| *Mus aP2* | GTGTGATGCCTTTGTGGGAAC | 235 | 60 |
|  | CCTGTCGTCTGCGGTGATT |  |  |
| *Mus Adiponectin* | GCTCTCCTGTTCCTCTTAATCCT | 437 | 60 |
|  | CCAGTGCTGCCGTCATAATG |  |  |
| *Mus Ebf1* | ACAAGCCACCAATCAAGG | 227 | 55 |
|  | GAAGGAGAAGATGCCAGAG |  |  |
| *Mus KLF4* | CCTTCGGTCATCAGTGTTA | 114 | 55 |
|  | CGCCTCTTGCTTAATCTTG |  |  |
| *Mus KLF5* | AACCAGACGGCAGTAATG | 254 | 55 |
|  | ATTGTAGCGGCATAGGAC |  |  |
| *Mus KLF15* | TACACCAAGAGCAGCCACCT | 110 | 55 |
|  | AACTCATCTGAGCGGGAAAAC |  |  |
| Mus *C/EBPα* | GGTTTCGGGTCGCTGGATCTCTAG | 151 | 60 |
|  | ACGGCCTGACTCCCTCATCTTAGAC |  |  |
| *Mus β-actin* | GGCACCACACCTTCTACAATG  GGGGTGTTGAAGGTCTCAAAC | 133 | 60 |

**Table S2**. Primers for the promoter truncation assay

| **Plasmid** | **Location** | **Vector** | **Primers (sense/antisense 5'-3')** | **RE site** |
| --- | --- | --- | --- | --- |
| P1 | -2501 ~ -47 | pGL3-basic | TC**GAGCTC**CACAATTCCTCGCCAA | SacI |
|  |  |  | CCG**CTCGAG**GCCAATCCATTAAAGG | XhoI |
| P2 | -646 ~ -47 | pGL3-basic | TC**GAGCTC**TCTCAGTCCATCCCACT | SacI |
|  |  |  | CCG**CTCGAG**GCCAATCCATTAAAGG | XhoI |
| P3 | -498 ~ -47 | pGL3-basic | TC**GAGCTC**CTTAGTAGGTTAAGGAT | SacI |
|  |  |  | CCG**CTCGAG**GCCAATCCATTAAAGG | XhoI |
| P4 | -301 ~ -47 | pGL3-basic | TC**GAGCTC**TGAACATGTGGGTCACT | SacI |
|  |  |  | CCG**CTCGAG**GCCAATCCATTAAAGG | XhoI |

RE, restriction enzymes; Restriction enzyme sites are underlined.


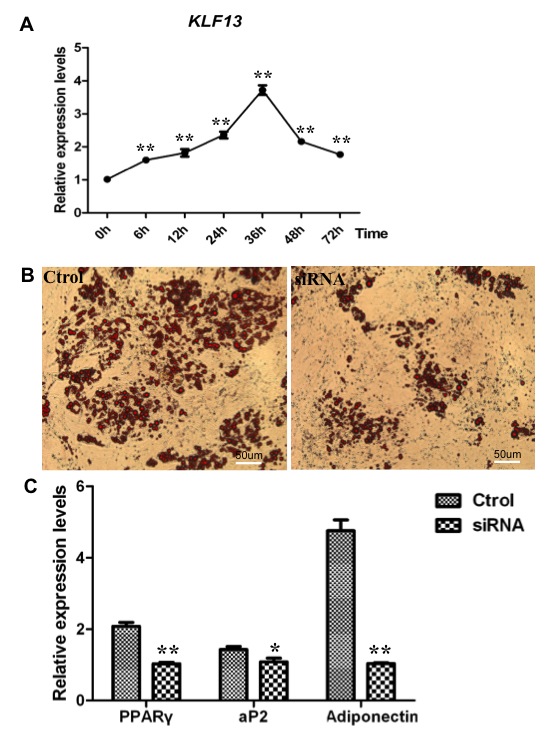


**Figure S1. Expression and function of KLF13 in porcine MASV**

(A) The mRNA expression of *KLF13* in porcine MSVC during adipocyte differentiation. The mRNA level was determined by real-time PCR and normalized to β-actin mRNA. The numbers indicate the time points of differentiation induction. Results are expressed as means ± SD. (n = 3) (B) Blocked MSVC adipocyte differentiation by KLF13 knockdown. MSVC were treated with KLF13 siRNA at about 70% confluence. After 24 h, the cells were induced to adipogenic differentiation. On day 8, the cell monolayer was stained with Oil-red O. (C) The mRNA expression of *PPARγ*, *aP2* and *Adiponectin* in KLF13-knockdown MSVC were detected by real-time PCR on day 8 after adipogenic induction. Results are expressed as means ± SD. (n = 3) **P*<0.05, ***P*<0.01


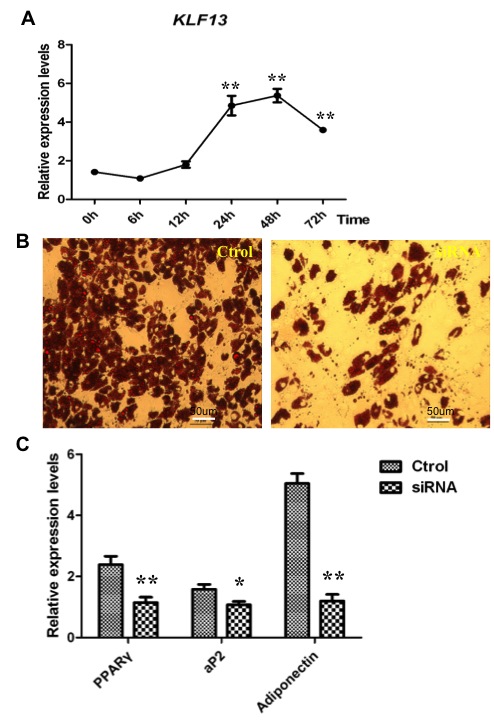

**Figure S2. Expression and function of KLF13 in porcine DFAT cells**

(A) The mRNA expression of *KLF13* in porcine DFAT cells during adipocyte differentiation. The mRNA level was determined by real-time PCR and normalized to β-actin mRNA. The numbers indicate the time points of differentiation induction. Results are expressed as means ± SD. (n = 3) (B) Blocked DFAT cells adipocyte differentiation by KLF13 knockdown. DFAT cells were treated with KLF13 siRNA at about 70% confluence. After 24 h, the cells were induced to adipogenic differentiation. On day 8, the cell monolayer was stained with Oil-red O. (C) The mRNA expression of *PPARγ*, *aP2* and *Adiponectin* in KLF13-knockdown DFAT cells were detected by real-time PCR on day 8 after adipogenic induction. Results are expressed as means ± SD. (n = 3) **P*<0.05, ***P*<0.01


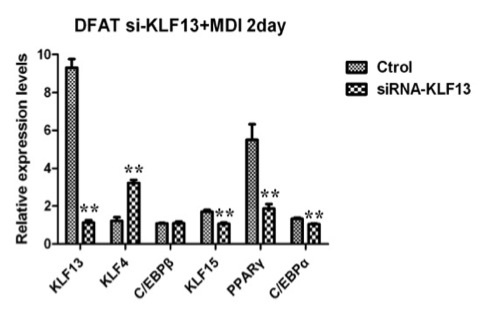


**Figure S3. Effect of knockdown KLF13 on the expression of adipogenic factors during adipogenic differentiation of porcine DFAT cells.**

After 1 days transfection of KLF13 siRNA, Porcine DFAT cells were stimulated in adipogenic induction medium for 2 days. Real-time PCR was used to determine the mRNA expression of *KLF13,* *KLF4, C/EBPβ, KLF15, PPARγ* and *C/EBPα*. Values are represented as mean ± SD. (n = 3) ***P*<0.01


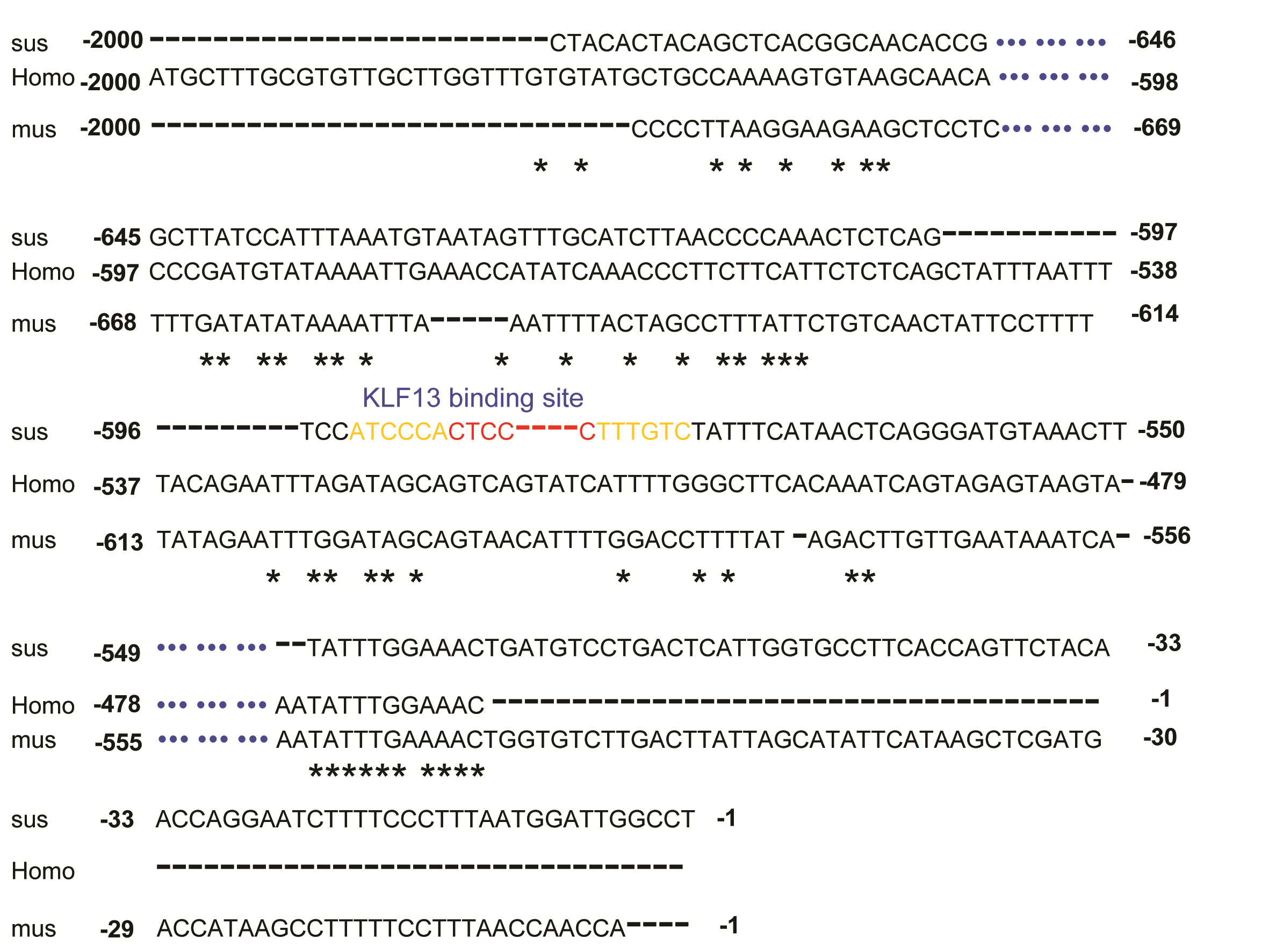


**Figure S4. Sequence of the promoter of the pig, human and mouse PPARγ2 genes.**

The DNA sequences of porcine PPARγ2 promoter (2000 bp), human PPARγ2 promoter (2000 bp) and mouse PPARγ2 promoter (2000 bp) were aligned.
